# Crosstalk of *Escherichia coli* FadR with Global Regulators in Expression of Fatty Acid Transport Genes

**DOI:** 10.1371/journal.pone.0046275

**Published:** 2012-09-28

**Authors:** Youjun Feng, John E. Cronan

**Affiliations:** 1 Department of Microbiology, University of Illinois, Urbana, Illinois, United States of America; 2 Department of Biochemistry, University of Illinois, Urbana, Illinois, United States of America; University of Florida, United States of America

## Abstract

*Escherichia coli* FadR plays two regulatory roles in fatty acid metabolism. FadR represses the fatty acid degradation (*fad*) system and activates the unsaturated fatty acid synthetic pathway. Cross-talk between *E. coli* FadR and the ArcA-ArcB oxygen-responsive two-component system was observed that resulted in diverse regulation of certain *fad* regulon β-oxidation genes. We have extended such analyses to the *fadL* and *fadD* genes, the protein products of which are required for long chain fatty acid transport and have also studied the role of a third global regulator, the CRP-cAMP complex. The promoters of both the *fadL* and *fadD* genes contain two experimentally validated FadR-binding sites plus binding sites for ArcA and CRP-cAMP. Despite the presence of dual binding sites FadR only modestly regulates expression of these genes, indicating that the number of binding sites does not determine regulatory strength. We report complementary *in vitro* and *in vivo* studies indicating that the CRP-cAMP complex directly activates expression of *fadL* and *fadD* as well as the β-oxidation gene, *fadH*. The physiological relevance of the *fadL* and *fadD* transcription data was validated by direct assays of long chain fatty acid transport.

## Introduction

**Figure 1:**
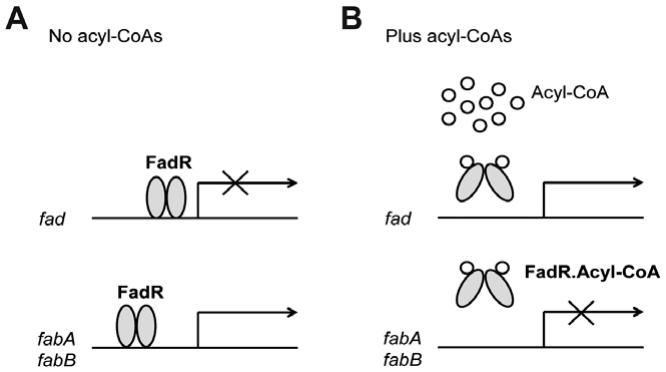
Regulation of fatty acid metabolism by *E. coli* FadR. ***A***. In the absence of a long chain acyl-CoA *E. coli* FadR represses the *fad* regulon genes [8], [23] whereas it activates transcription of *fabA* and *fabB*, the two genes of the unsaturated fatty acid synthetic pathway [7]–[9]. ***B***. Binding of long chain acyl-CoA species results in large changes in FadR structure resulting in dissociation of the protein from its operator sites. Dissociation increases *fad* regulon expression whereas expression of *fabA* and *fabB* is decreased. The ovals denote FadR whereas the small open circles represent the acyl-CoA pool.

Much of our current knowledge of bacterial fatty acid metabolism comes from studies with *Escherichia coli* [1]. The fatty acid degradation (*fad*) pathway is primarily responsible for the transport, activation and β-oxidation of fatty acids [2]. The known long-chain fatty acid (LCFA) transport system components are the outer-membrane transport protein, FadL [3], [4] and the inner-membrane associated acyl-CoA synthetase, FadD [5], [6]. In this system, the FadL transporter delivers exogenous long chain fatty acids across the cell membrane into the periplasmic space [3], [4]. From there by an unknown mechanism the LCFA enter the cytosol where they become activated to their acyl-CoA thioesters by FadD which allows entry of the acyl chains into the β-oxidation cycle [5], [6]. *E. coli* FadR coordinates the catabolic and anabolic fatty acid pathways (Fig. 1). In this dual role FadR acts as a repressor for the entire set of *fad* regulon genes and also functions as an activator for unsaturated fatty acid biosynthesis pathway by increasing transcription of both *fabA* [7], [8] and *fabB* [9] (Fig. 1). The physiological ligands that antagonize FadR binding to its cognate operators are LCFA CoA thioesters synthesized by the cytosolic acyl-CoA synthetase, FadD [7], [10]. In strains lacking either FadD or FadL addition of LCFA fails to induce *fad* regulon expression because the regulatory ligand cannot be synthesized. Therefore, extremely stringent regulation of *fadD* and *fadL* genes is precluded because this would block derepression.

Similar to most of the other *fad* regulon genes, *fadL* [4], [11], [12] and *fadD* [12], [13] are controlled at the transcriptional level by two different regulatory systems in addition to FadR, the oxygen-sensitive ArcA-ArcB two-component system and the cyclic AMP (cAMP) receptor protein-cyclic AMP (CRP-cAMP) complex. Our interest in *fadL* and *fadD* arose from the presence of two FadR-binding sites in the promoters of both genes whereas the other FadR regulon *fad* genes each have only a single site. This raised the question of regulatory interactions among the multiple regulators, FadR, ArcA and CRP-cAMP when bound to the respective promoter regions. Cho and coworkers [12] initially used quantitative RT-PCR to assay regulation of *fadL* and *fadD* by FadR and ArcA under anaerobic conditions and reported that deletion of either *arcA*, *fadR* alone, or both *arcA* and *fadR* resulted in increased *fadL* expression by 34-, 4- or 69-fold, respectively, whereas *fadD* transcription increased 69-, 4.5-, and 84-fold. These workers did not investigate the effects of CRP-cAMP. Classical catabolite repression of the *fad* pathway has long been known. Pauli and coworkers [14] reported that Fad enzyme levels were very low in wild type cells grown in glucose even in the presence of inducing levels of fatty acid (or in constitutive *fadR* mutant strains) and that glucose inhibition could be partially relieved by addition of cAMP. CRP mutant strains also had low *fad* enzyme activities. In this laboratory Clark [15] using early *lacZ* fusion technology showed that glucose acted at the transcriptional level in *fadBA* and *fadE* expression. Although as discussed below CRP-cAMP binding sites for several *fad* genes have been proposed, none had been directly tested for the ability to bind the complex. Therefore, it remained possible that CRP-cAMP regulation of the *fad* regulon was an indirect effect.

We report experiments defining the interactions of these regulatory proteins and their effects on fatty acid transport.

## Materials and Methods

### Bacterial strains and growth conditions

All the strains used here are *E. coli* K-12 derivatives (Table 1). The bacterial media were used for bacterial growth and analyses of β-galactosidase activity. These included LB medium (Luria-Bertani medium containing 10 g of tryptone, 5 g of yeast extract and 10 g of NaCl per liter), Rich broth (RB medium containing 10 g of tryptone, 1 g of yeast extract, and 5 g of NaCl per liter) and minimal medium M9 [16] supplemented with 0.4% glucose or other carbon source, 0.1% Vitamin-Free Casamino Acids, 0.1 mM CaCl_2_, and 0.001% thiamine. When necessary, antibiotics were used as follows (in mg/liter): sodium ampicillin, 100; kanamycin sulfate, 50; chloramphenicol, 20 and tetracycline HCl, 15. To monitor transcriptional regulation by ArcA-ArcB, an anaerobic environment (5% H_2_, 75% N_2_, and 20% CO_2_) was generated in an anaerobic environmental system (Bio-Bag environmental chamber type A; Becton Dickinson). The *E. coli* strains were grown on modified LB media in which potassium nitrate (5 mM) was added as an electron acceptor [17]. Fatty acids such as oleic acid (Sigma) were neutralized with KOH, solubilized with Tergitol NP-40, and used as an inducer at 5 mM final concentration.

**Table 1:** Strains and plasmids used in this study.

Bacteria or plasmids	Relevant characteristics	References/sources
***E. coli*** ** strains**		
Topo10	F^−^, Δ*lac*X74, a cloning *h*ost for recombinant plasmids	Invitrogen
BL21(DE3)	Protein expression host	Lab collection
MG1655	Wild type of *E. coli* K-12	CGSC^a^, Lab collection
MC4100	F^−^, *araD*139, Δ(*argF-lac*)169	[25]
DH5α (λ-*pir*)	Δ*lac* host for pAH125 and its derivatives	[25], [37]
MC1061	Wild type of *E. coli* K-12, Δ*lac*	[23]
MFH8	UB1005, *fadR*::Tn*10*	[7]
BW25113	A wild type strain of *E. coli* K-12	CGSC^a^, [38]
JW2341-1	F^−^ Δ(*araD-araB*)*567* Δ*lacZ4787*(::rrnB-3) *LAM-* Δ*fadL752:: kan rph-1* Δ (*rhaD-rhaB*) *568 hsdR514*	CGSC^a^, [38]
JW1794-1	F^−^ Δ(*araD-araB*)*567* Δ*lacZ4787*(::rrnB-3) *LAM-* Δ*fadD730::kan rph-1* Δ (*rhaD-rhaB*) *568 hsdR514*	CGSC^a^, [38]
FYJ55	*fadH-lacZ* transcriptional fusion	[17]
FYJ56	*fadH-lacZ* transcriptional fusion, Δ*fadR*::Tn*10*	[17]
FYJ57	JW1176-1, Δ*fadR*	[17]
FYJ59	JW4361-1, Δ*arcA*	[17]
FYJ60-2	JW5702-4, Δ*crp*	[17]
FYJ65	*fadH-lacZ* transcription fusion, Δ*crp*	[17]
FYJ76	FYJ60-2, Δ*crp* Δ*arcA726::kan*	[17]
FYJ77	FYJ76, Δ*arcA* Δ*crp*	[17]
FYJ78	FYJ77, Δ*arcA* Δ*crp* Δ*fadR::*Tn*10*	[17]
FYJ82	*fadBA-lacZ* transcriptional fusion, Δ*arcA*	[17]
FYJ83	*fadBA-lacZ* transcriptional fusion, Δ*crp*	[17]
SI203	*fadBA-lacZ* transcriptional fusion	[17], [39]
SI207	*fadBA-lacZ* transcriptional fusion, Δ*fadR::*Tn*10*	[17], [39]
FYJ103	JW2341-1 carrying pCP20*ts*, Δ*fadL*	This work
FYJ104	FYJ103, *fadL-lacZ* transcriptional fusion	This work
FYJ105	FYJ104, Δ*fadR::*Tn*10*, *fadL-lacZ* transcription fusion	*P1_vir_*(MFH8)×FYJ104^b^
FYJ118	MC1061, Δ*fadL::km*	This work
FYJ119	FYJ118 carrying pCP20, Δ*fadL*	This work
FYJ132	JW1116-1 carrying pCP20ts	This work
FYJ133	FYJ59, Δ*arcA*, *fadL-lacZ* transcriptional fusion	P1*vir*(FYJ104)×FYJ59^c^
FYJ134	FYJ60-2, Δ*crp*, *fadL-lacZ* transcriptional fusion	P1*vir*(FYJ104)×FYJ60-2^c^
FYJ139	FYJ59, Δ*arcA* Δ*fadR::*Tn*10*	P1*_vir_*(MFH8)×FYJ59^b^
FYJ140	FYJ60-2, Δ*crp* Δ*fadR::*Tn*10*	P1*_vir_*(MFH8)×FYJ60-2^b^
FYJ141	FYJ139, Δ*arcA* Δ*fadR::*Tn*10*, *fadL-lacZ* transcriptional fusion	P1*_vir_*(FYJ104)×FYJ139^c^
FYJ142	FYJ140, Δ*crp* Δ*fadR::*Tn*10*, *fadL-lacZ* transcriptional fusion	P1*_vir_*(FYJ104)×FYJ140^c^
FYJ158	DH5α (λ-*pir*) carrying pAH-*PfadD*	[22]
FYJ159	MC4100 with *fadD-lacZ* transcriptional fusion at the chromosomal *attB*_λ_ site.	[22]
FYJ161	FYJ57, Δ*fadR, fadD-lacZ* transcriptional fusion	P1*vir*(FYJ159)×FYJ57^c^
FYJ162	FYJ59, Δ*arcA, fadD-lacZ* transcriptional fusion	P1*vir*(FYJ159)×FYJ59^c^
FYJ163	FYJ60-2, Δ*crp, fadD-lacZ* transcription fusion	P1*vir*(FYJ159)×FYJ60-2^c^
FYJ164	FYJ139, Δ*arcA* Δ*fadR::*Tn*10*, *fadD-lacZ* transcriptional fusion	P1*vir*(FYJ159)×FYJ139^c^
FYJ165	FYJ140, Δ*crp* Δ*fadR::*Tn*10*, *fadD-lacZ* transcriptional fusion	P1*vir*(FYJ159)×FYJ140^c^
FYJ166	FYJ77, Δ*crp* Δ*arcA*, *fadD-lacZ* transcription fusion	P1*vir*(FYJ159)×FYJ77^c^
FYJ167	FYJ78, Δ*crp* Δ*arcA* Δ*fadR::*Tn*10*, *fadD-lacZ* transcription fusion	P1*vir*(FYJ159)×FYJ78^c^
FYJ169	FYJ77, Δ*crp* Δ*arcA*, *fadL-lacZ* transcriptional fusion	P1*vir*(FYJ104)×FYJ77^c^
FYJ170	FYJ78, Δ*crp* Δ*arcA* Δ*fadR::*Tn*10*, *fadL-lacZ* transcriptional fusion	P1*vir*(FYJ104)×FYJ78^c^
FYJ187	MC4100 carrying pINT-ts	[22]
FYJ238	Topo 10 carrying pET28-*crp*	This work
FYJ239	BL21(DE3) carrying pET28-*crp*	This work
FYJ294	DH5α (λ-*pir*) carrying pAH-*PfadL*	This work
FYJ295	MC4100 with *fadL-lacZ* transcriptional fusion at the chromosomal *attB*_λ_ site.	This work
**Plasmids**		
pET28(a)	T7 promoter expression Km^R^ vector	Novagen
pKD46	*bla* P_BAD_ *gam* pSC101, oriTS, Amp^R^	[40]
pKD13	*bla* FRT *ahp* FRT PS1 PS4 oriR6K, Km^R^	[40]
pCP20	*bla cat cI*857 *λP*_R_ *flp* oriTS, Amp^R^, Cm	[19], [41]
pKG137	Transcription fusion plasmid, *ahp* FRT *lacZY* ^+^ t_his_ oriR6K, Km^R^	[19], [40]
pINT-ts	Temperature sensitive helper plasmid expressing Int_λ_, Amp^R^	[37]
pAH125	A promoter-less *lacZ* reporter plasmid in *E. coli*, Kan^R^	[25], [37]
pAH-*PfadD*	pAH125 carrying *fadD* promoter region, Kan^R^	[22]
pAH-*PfadL*	pAH125 carrying *fadL* promoter region, Kan^R^	This work
pET28-*fadR*ec	pET28 carrying wild type *fadR*	[23], [25]
pET28-*crp*	pET28 encoding CRP	This work

a CGSC denotes Coli Genetic Stock Center, Yale University.

b Selection for tetracycline resistance.

c Selection for kanamycin resistance.

### Plasmids and genetic manipulations

The pCR2.1-TOPO vector (Invitrogen) was used for PCR cloning and sequencing whereas expression vector pET28a (Novagen) was used for protein preparation (Table 2). All plasmids constructed were validated by PCR analyses plus direct DNA sequencing.

**Table 2:** Primers used in this study.

Primers	Primer sequences (5′–3′)
*fadL*-PF	CGT TGA TTT CCT CTG TAT GTG C
*lacZ-R*	GAC CAT GAT TAC GGA TTC ACT G
*fadL*-promoter-F (SalI)	CCG *GTCGAC* CGT TGA TTT CCT CTG TAT GTG
*fadL*-promoter-R (EcoRI)	AACC *GAATTC* GCG AGA GCA GAC TTT GTA AAC
*fadD*-promoter-F (SalI)	CCG *GTCGAC* GTT GCG GTA CAA AAC CAG CA
*fadD*-promoter-R (EcoRI)	AACC *GAATTC* CTC TAA AAT GCG TGT TCG TCG
*crp*-F1 (BamHI)	CG *GGATCC* ATG GTG CTT GGC AAA CCG CA
*crp*-R1 (XhoI)	CCG *CTCGAG* TTA ACG AGT GCC GTA AAC GAC
*fadL*-FadR-site1-F	GCA ACA TTC C**AG CTG GTC CGA CCT ATA** CTC TCG CC
*fadL*-FadR-site1-R	GGC GAG AG**T ATA GGT CGG ACC AGC T**GG AAT GTT GC
*fadL*-FadR-site2-F	CTC TCG CC**A CTG GTC TGA TTT CTA A**GA TGT ACC TC
*fadL*-FadR-site2-R	GAG GTA CAT C**TT AGA AAT CAG ACC AGT** GGC GAG AG
*fadD*-FadR-site1-F	GTA ATT ATC A**AG CTG GTA TGA TGA GTT** AAT ATT ATG
*fadD*-FadR-site1-R	CAT AAT ATT **AAC TCA TCA TAC CAG CT**T GAT AAT TAC
*fadD*-FadR-site2-F	GAA ACA GC**G GCT GGT CCG CTG TTT C**TG CAT TCT
*fadD*-FadR-site2-R	AGA ATG CA**G AAA CAG CGG ACC AGC C**GC TGT TTC
*fadD*-CRP-site-F	GTA AAG ATA AAA ATA **AAT AGT GAC GCG CTT CGC AAC C**TT TTC GTT GGG
*fadD*-CRP-site-R	CCC AAC GAA AA**G GTT GCG AAG CGC GTC ACT ATT** TAT TTT TAT CTT TAC
*fadL*-CRP-site1-F	CTG CAA AAT CGG **ATA AGT GAC CGA AAT CAC ACT T**AA AAA TGA TCT
*fadL*-CRP-site1-R	AGA TCA TTT TT**A AGT GTG ATT TCG GTC ACT TAT** CCG ATT TTG CAG
*fadL*-CRP-site2-F	CCC TAC ACT TCG CGC **TCC TGT TAC AGC ACG TAA CAT A**GT TTG TAT AAA AAT AAA TC
*fadL*-CRP-site2-R	GAT TTA TTT TTA TAC AAA C**TA TGT TAC GTG CTG TAA CAG GA**G CGC GAA GTG TAG GG
*fadH*-CRP-site1-F	GTA ACC TGG ATA **AAA CGC GAC AAG CGT CGC ATC C**GG CGT TAT CAC CGG
*fadH*-CRP-site1-R	CCG GTG ATA ACG CC**G GAT GCG ACG CTT GTC GCG TTT** TAT CCA GGT TAC
*fadH*-CRP-site2-F	CAC CGG GCG TAT **TCT TTT TGA ATC CCA TCA CAA A**CC CCG CAC TCC
*fadH*-CRP-site2-R	GGA GTG CGG GG**T TTG TGA TGG GAT TCA AAA AGA** ATA CGC CCG GTG
T7-F	TAA TAC GAC TCA CTA TAG GG
T7-R	GCT AGT TAT TGC TCA GCG G

The sequences underlined are restriction sites, and the bold letters are predicted FadR or the CRP-cAMP sites.

Both FYJ159 and FYJ295 are, respectively, derivatives of *E. coli* strain MC4100, in which either a *fadD–lacZ* transcriptional fusion [18] or a *fadL-lacZ* transcriptional fusion, was integrated into the chromosomal *att*λ site (Table 1). Strain FYJ104 which carries a chromosomal *fadL-lacZ* transcription fusion (Table 1) was constructed using the FLP-mediated site-specific recombination method [19]. The kanamycin resistance cassette was removed from the *fadL*::km strain JW2341-1 (Table 1) by expression of the FLP recombinase encoded by plasmid pCP20 (Table 1) to give strain FYJ103, which retained a single FLP recombinase target (FRT) site. The FRT site was used for site-specific integration of the *lacZ* fusion plasmid, pKG37 (an improved version of pCE71) containing a FRT site upstream of a promoterless *lacZY* genes, a kanamycin resistance gene, and the R6K origin of replication (Table 1) [19]. The transformants were screened on LB agar plates containing kanamycin and 5-bromo-4-chloro-3-indolyl-β-D-galactopyranoside (X-Gal) at 37°C to obtain the chromosomal *fadL-lacZ* fusion strain FYJ104. The fusion plasmid was stably integrated due to its R6K origin and loss of the temperature-sensitive pCP20 plasmid [19]. The *fadL* promoter-*lacZ* junction was validated by a PCR using primers, *fadL*-P plus *lacZ*-R (Table 2).

### P1_vir_ phage transduction

P1*_vir_* transductions were carried out as described by Miller [20] with minor modifications. Strains FYJ133, FYJ134, FYJ141, FYJ142, FYJ169 and FYJ170 were generated by transduction of strains FYJ59 (Δ*arcA*), FYJ60-2 (Δ*crp*), FYJ139 (Δ*arcA* Δ*fadR*::Tn*10*), FYJ140 (Δ*crp* Δ*fadR*::Tn*10*), FYJ77 (Δ*crp* Δ*arcA*), and FYJ78 (Δ*crp* Δ*arcA* Δ*fadR*::Tn*10*), respectively (Table 1) with a P1*_vir_* lysate grown on FYJ104 (*fadL-lacZ*) with selection for kanamycin resistance. Similarly, a *P1_vir_* lysate grown on FYJ159 (*fadD-lacZ*) was used for transduction of the following strains FYJ57, FYJ59, FYJ60-2, FYJ139, FYJ140, FYJ77 and FYJ80 with selection for kanamycin resistance to give strains FYJ161, FYJ162, FYJ163, FYJ164, FYJ165, FYJ166 and FYJ167, respectively (Table 1). Transduction of strains FYJ104, FYJ59 and FYJ60-2 with a *P1_vir_* lysate grown on MFH8 (*fadR*::Tn*10*) with selection for tetracycline resistance gave strains FYJ105 (Δ*fadR*::Tn*10*, *fadL-lacZ*), FYJ139 (Δ*arcA* Δ*fadR*::Tn*10*) and FYJ140 (Δ*crp* Δ*fadR*::Tn*10*), respectively.

### β-Galactosidase assays

Mid-log phase cultures in LB, RB or minimal media (with or without supplementation with various carbon sources), were collected by centrifugation, washed twice with Z Buffer [21] and assayed for β-galactosidase activity after lysis with sodium dodecyl sulfate-chloroform [21]. The data were recorded in triplicate with no less than three independent experiments.

### Protein expression and purification

Hexahistidine-tagged *E. coli* FadR (and/or CRP) proteins were produced in *E. coli* BL21 (DE3) carrying the expression plasmid pET28-*fadR*ec (and/or pET28-*crp*) (Table 1) by induction of bacterial cultures at an OD_600 nm_ of 0.8–1.0 with 0.3 mM IPTG at 30°C for 3 h [17], [22]. The cells were pelleted washed twice with ice cold PBS buffer (101.4 mM Na_2_HPO_4_, 1.8 mM KH_2_PO_4_, 137 mM NaCl, 2.7 mM KCl, 8% glycerol, pH 7.4), dissolved in the same buffer and lysed using a French pressure cell. The extracts were centrifuged to remove bacterial debris and the supernatants loaded onto a nickel chelate column (Qiagen). Following washing with ten column volumes of with PBS buffer containing 50 mM imidazole, the FadR proteins were eluted with 150 mM imidazole. Appropriate eluted protein fractions were pooled and dialyzed against PBS buffer then concentrated by ultrafiltration (30 kDa cut-off, Amicon Ultra) [17]. The protein purity was judged by 12% SDS-PAGE, followed by staining with Coomassie brilliant blue R250 (Sigma, St. Louis, MO). Both FadR and CRP proteins were characterized by liquid chromatography quadrupole time-of-flight mass spectrometry of tryptic peptides and chemical cross-linking as described previously [18].

### Electrophoretic mobility shift assays

These assays of the interaction between the *fadD* and *fadL* promoters, FadR and the cAMP-CRP complex were done essentially as previously reported [18], [23]. All of the FadR (and/or CRP) probes were prepared by annealing two complementary primers (Table 2) by incubation in TEN buffer (10 mM Tris-HCl, 1 mM EDTA, 100 mM NaCl, pH 8.0) at 95°C for 5 min followed by slow cooling to 25°C and then DIG labeling by terminal transferase with DIG-ddUTP (Roche). DNA probes (Table 2) for assay of DNA binding by the CRP-cAMP complex were similarly synthesized. The digoxigenin-labeled DNA probes (either 0.1 or 0.2 pmol) were incubated with either DNA binding protein in binding buffer (Roche) for 15 min at room temperature and then analyzed by native PAGE (6.5% PAGE for the CRP probes and 7% PAGE for all other probes). The separations were then visualized as previously described [18], [23].

### Fatty acid transport assays

Fatty acid transport was assayed as described by Klein *et al.* [24] with minor modifications. To avoid complications by β-oxidation strains that carried a *fadBA* disruption (strains FYJ82, FYJ83 and SI203) were used (Table 1). To test ArcA-P regulation of fatty acid transport, FYJ82 strain (Δ*arcA*) was compared with the wild type strain SI203 (Table 1). Overnight cultures were inoculated into 10 ml of RB liquid media supplemented with potassium nitrate (5 mM) as electron acceptor and kept in a fully anaerobic chamber at 37°C for ∼10 hrs [17]. The anaerobic environment (5% H_2_, 75% N_2_, and 20% CO_2_) was generated by an anaerobic environmental chamber (Bio-Bag type A, Becton Dickinson) [17]. Cultures in exponential phase were treated with 100 mM chloramphenicol for 10 min prior to assay. 1-^14^C-Oleic acid (American Radio-labeled Chemicals) was injected into the anaerobic bacterial cultures using a syringe fitted with a fine needle (30G1 PrecisionGlide) to a final concentration of 45.5 µM. The cultures were mixed well by vigorous vortex mixing and incubated at room temperature for about 15 min with anaerobiosis monitored by an anaerobic indicator (0.001% resazurin). Finally, the bacteria were collected by centrifugation (4.200× *g*, 16 min), and washed for five times with iced RB medium. One ml of the cultures were subjected to membrane phospholipid extraction [18], [25] after measuring culture absorbance (A_600_) and adjusted to an absorbance of 1.5. The phospholipids were then separated from any residual fatty acid by thin layer chromatography [18], [25]. Assay of the effects of the CRP-cAMP system on logarithmic phase cultures aerobically grown in LB liquid proceeded in a similar manner.

### Bioinformatic analyses

The known or predicted DNA binding sites recognized by either FadR or ArcA (or CRP) were all from the *E. coli* literature. Multiple alignments were done using ClustalW2 (http://www.ebi.ac.uk/Tools/clustalw2/index.html), and the resultant output was processed by program ESPript 2.2 (http://espript.ibcp.fr/ESPript/cgi-bin/ESPript.cgi), generating the final BLAST version.

## Results and Discussion

### Regulatory complexity in the LCFA transport promoters, *fadL* and *fadD*

**Figure 2:**
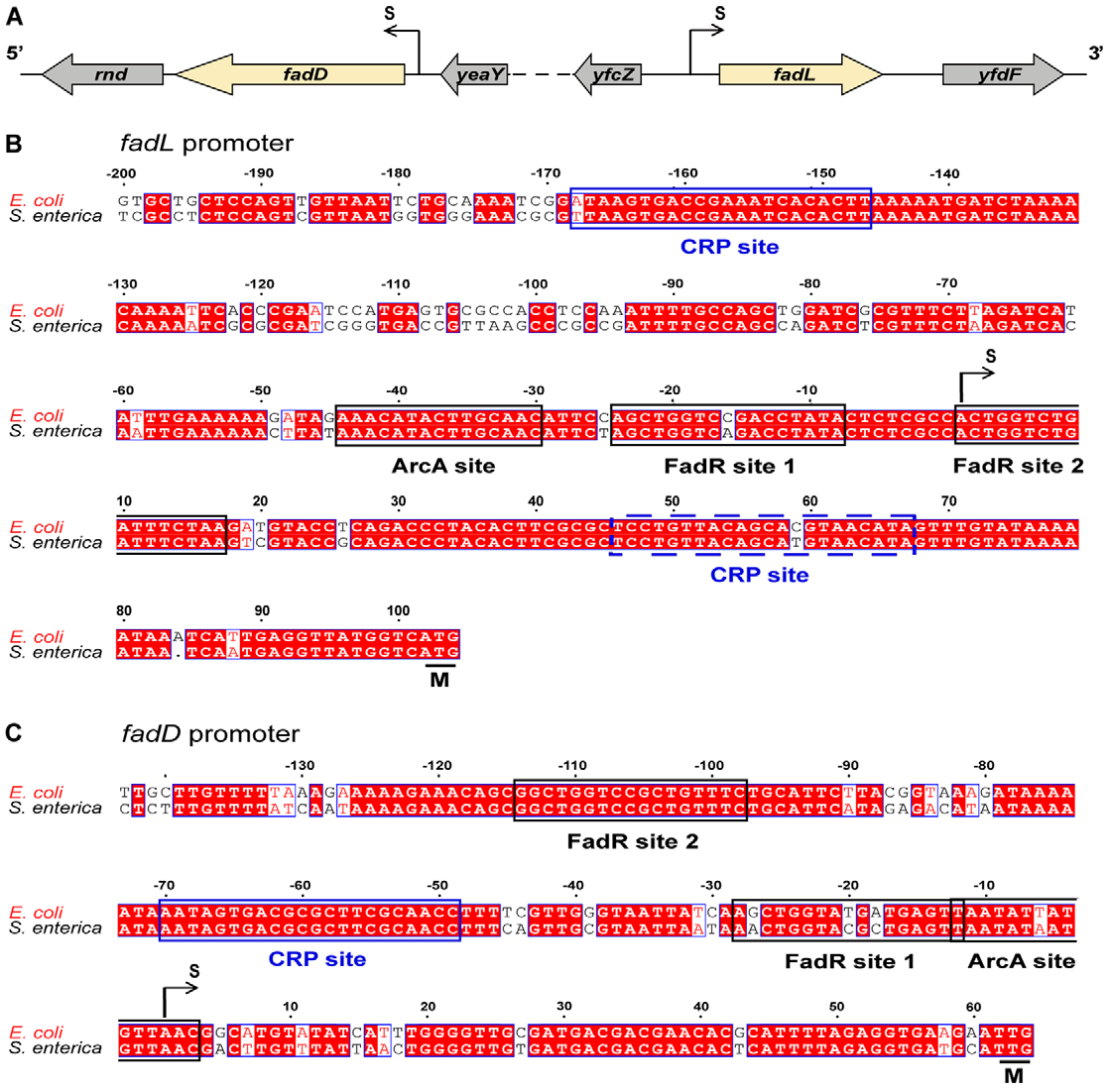
The *fadL* and *fadD* promoters of *E. coli*. ***A***. Genomic contexts of *fadL* and *fadD* on the *E. coli* chromosome (the two genes are separated by 570 kb). The transcription start sites (S) are indicated by arrows. Dotted lines denote spanning with long distance. ***B*** and ***C***. Sequences of the *fadL* promoter and the *fadD* promoter, respectively. All the demonstrated sites bound by either FadR or ArcA are labeled with black rectangles, whereas the annotated CRP binding sites that were validated in this study are labeled with blue rectangles. A dotted rectangle labels a *fadL* CRP binding site incorrectly predicted by EcoCyc (http://www.ecocyc.org). White letters shaded in red denote the identical residues, red letters shaded in white mean similar bases, whereas white-shaded black letters denote unrelated. Dots denote gaps. “M” denotes the translation initiation site. The numbers are given relative to the transcription start site (S). The *fadL* and *fadD* FadR binding sites and their relative spacing are largely conserved in the genomes of *Citrobacter*, *Kelbsiella* and *Enterobacter*.

The *fadL* and *fadD* genes encode the proteins known to be required for LCFA transport (Fig. 2A). The *fadL* and *fadD* transcriptional start sites are located 95 bp and 60 bp upstream of the translation start sites, respectively [6], [26] (Fig. 2B). Despite a seemingly straightforward role in metabolism, both promoters contain demonstrated or annotated binding sites for three different transcription factors, FadR, ArcA and CRP (http://www.ecocyc.org). Moreover, *fadL* and *fadD* promoters are the only *fad* regulon promoters that contain two distinct FadR binding sites [6], [26]. In the *fadL* promoter the FadR sites are separated by only 8 bp whereas in the *fadD* promoter the two FadR sites are separated by 68 bp (Fig. 2B and C). The locations of the ArcA binding sites determined by Cho and coworkers [12] also differ. The *fadL* ArcA site is 5 bp from FadR site 1 whereas the *fadD* ArcA site overlaps FadR site 2 by one bp, raising the possibility of crosstalk between the two repressor proteins (Fig. 2). The *fadL* CRP site was reported to lie downstream of the transcription start site [4] (Fig. 2B), a position incompatible with the usual activator function of CRP. Searches using the CRP consensus sequence of Zheng *et al.* [27] produced another *fadL* candidate binding site upstream of the transcription start site. Although this seemed a much more plausible position for activation of transcription, experimental verification of the CRP binding site was required. Indeed, although CRP-cAMP regulation of the *fad* regulon genes at the physiological level has been known for many years [14], none of the proposed sites have been experimentally validated.

### The two FadR-binding sites of *fadL* and *fadD* result in only modest repression

**Figure 3:**
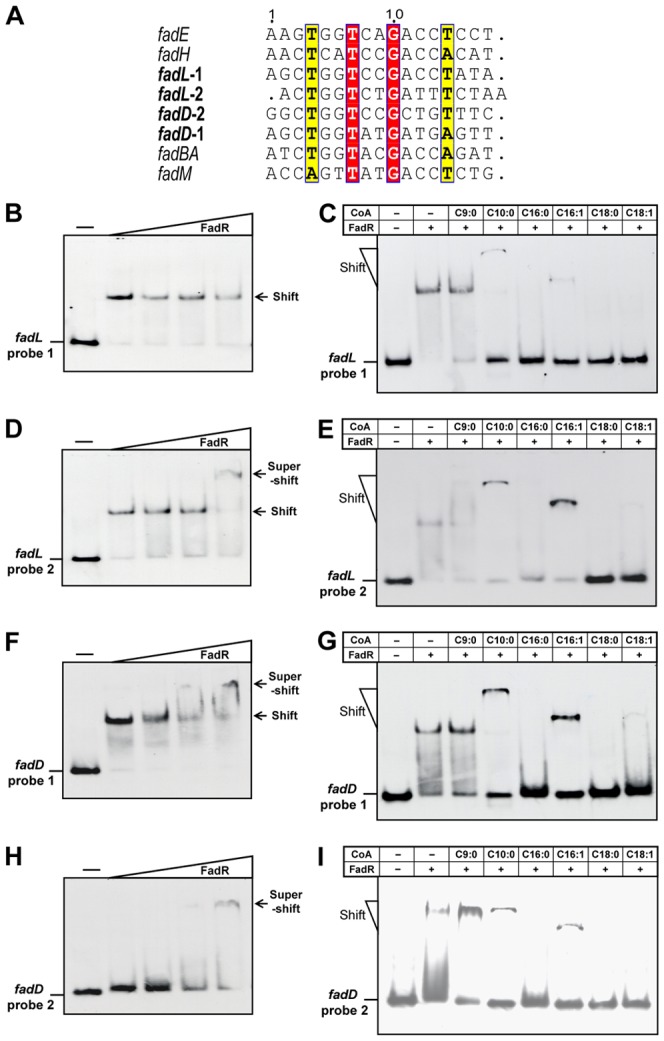
*E. coli fadL* and *fadD* both carry two functional FadR-binding sites. ***A***. sequence alignments of several known FadR binding sites from *E. coli fad* regulon. White letters with red background denote strictly conserved bases whereas yellow background letters denote highly conserved residues. The dual FadR sites of *fadL and fadD* are highlighted in bold italics. ***B*** and ***D***. Gel shift assays of FadR binding to both *fadL* promoter sites. ***F*** and ***H***. Gel shift assays of FadR binding to both *fadD* promoter sites. Long chain fatty acyl-CoA species block binding of FadR to the two *fadL* sites (***C*** *and* ***E***), as well as to the two *fadD* sites (***G*** *and* ***I***). FadR was used at concentrations of 0 (denoted by a minus sign), 1, 2, 5, or 10 pmol. FadR was incubated with 0.1 pmol of DIG-labelled probe in a total volume of 10 µl. For the acyl-CoA experiments the components were: probe, 0.1 pmol; FadR, 1 pmol and acyl-CoA, 50 pmol. Designations: C9:0; nonanoyl–CoA; C10:0, decanoyl-CoA; C16:0, palmitoyl-CoA; C16:1, palmitoleoyl-CoA; C18:0, stearoyl-CoA; C18:1, oleoyl-CoA.

**Figure 4:**
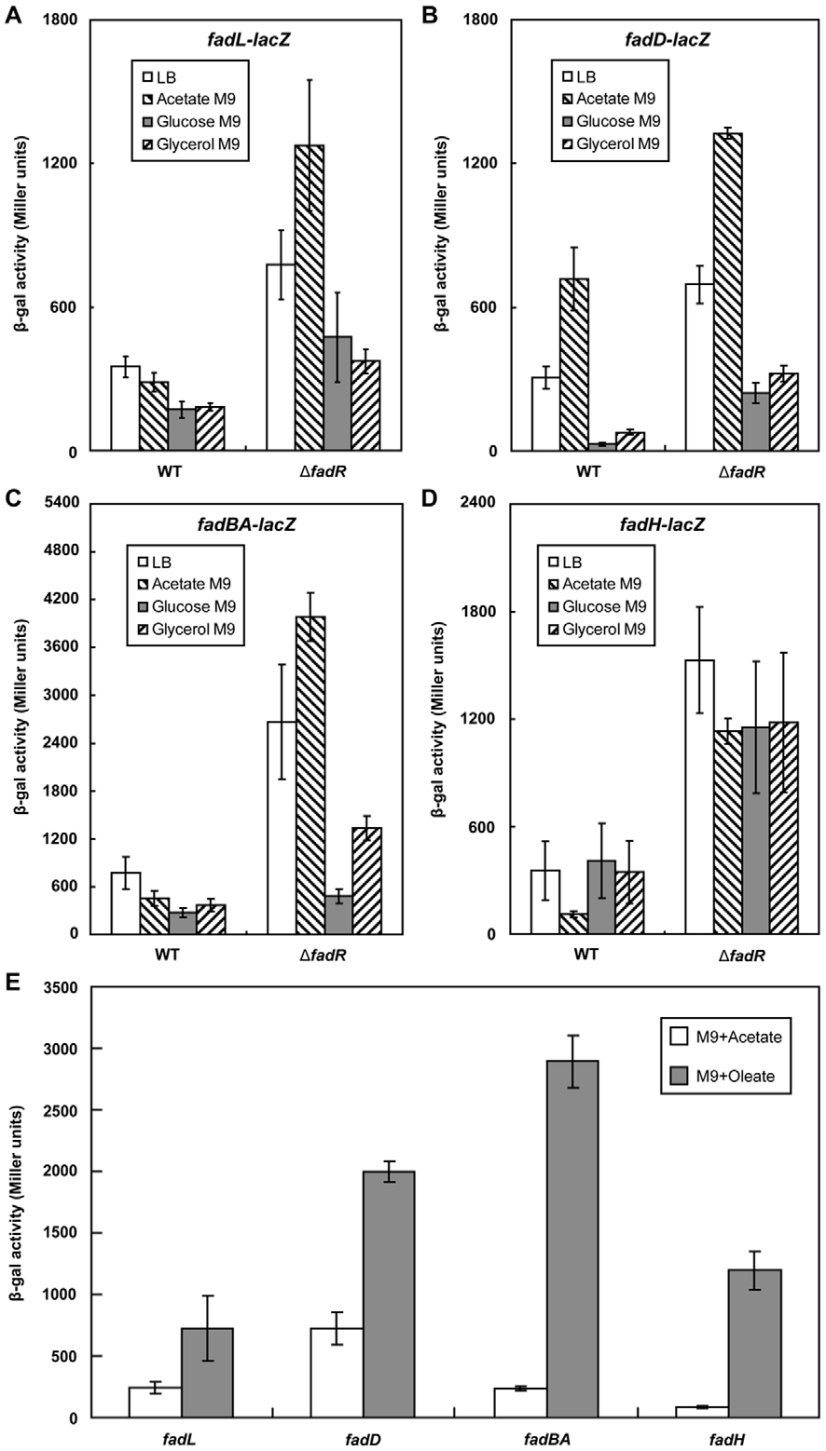
FadR repression and oleate induction of fatty acid transport system. ***A*** and ***B***. Repression of *fadL* and *fadD* by FadR in cultures grown on various carbon sources. ***C*** and ***D***. Parallel experiments with two β-oxidation genes *fadBA* and *fadH* are given for comparison. Strains FYJ104 (wild type) FYJ105 (Δ*fadR*::Tn*10*) were used for *fadL* whereas strains FYJ159 (wild type) and FYJ161 (Δ*fadR*) were used for *fadD*. Strains SI203 (wild type) and SI207 (Δ*fadR*) were used for *fadBA* whereas strains FYJ55 (wild type) and FYJ56 (Δ*fadR*) were used for *fadH*. ***E***. Induction of *fadL* and *fadD* expression by oleate. Oleate induction of the fatty acid transport system genes was compared with those of both *fadBA* and *fadH* expression. Oleic acid (5 mM) was added as the sole carbon source and compared to 5 mM acetate. Four strains expressing wild type FadR (FYJ295 (*fadL-lacZ*), FYJ159 (*fadD-lacZ*), SI203 (*fadBA-lacZ*), and FYJ55 (*fadH-lacZ*), were used. All strains were grown under aerobic condition (10 ml of culture in a 50 ml flask shaken at 200 rpm at 37°C). ß -Galactosidase (ß-gal) assays were conducted in triplicate and the error bars indicate standard deviations.

To obtain a parallel comparison of the FadR binding sites of the two promoters we used electrophoretic mobiltity shift analyses (EMSA) to assay the binding abilities of the individual sites over a range of FadR concentrations (Fig. 3). The two *fadL* FadR sites bound FadR with equivalent affinities (essentially complete binding of the probes by 50 nM FadR, Fig. 3B and D). In contrast, although *fadD* site 1 showed a binding affinity comparable to that of the *fadL* sites (Fig. 3F), FadR binding by *fadD* site 2 (Fig. 3H) was >10-fold weaker than the other three sites (Fig. 3B, D and F). Addition of 25–50 µM oleoyl-CoA resulted in loss of FadR binding by all four sites (Fig. 3C, E, G and I). Therefore, our data are in good agreement with the reported DNase I foot-printing results [6], [11] and provide data on the effects of the acyl-CoA regulatory ligand that is lacking in the foot-printing experiments. To monitor expression of *fadL* and *fadD*, each promoter was fused to a LacZ reporter gene to allow expression to be assayed by β-galactosidase activity (Fig. 4). Deletion of FadR resulted in only modest derepression of the two LCFA transport system genes (2 to 2.5 fold for *fadL* and 2 to 3-fold for *fadD*) in medium with acetate as sole carbon source (Fig. 4A and B) whereas expression of *fadBA* and *fadH* increased by 5 to10-fold (Fig. 4C and D). Increased expression of genes *fadL* and *fadD* in the Δ*fadR* strain was also seen when the carbon source was either glucose or glycerol (Fig. 4A and B), These observations are generally consistent with those obtained upon oleate induction of strains carrying a functional FadR (Fig. 4E).

### Expression of *fadL and fadD* is directly activated by the CRP-cAMP complex

**Figure 5:**
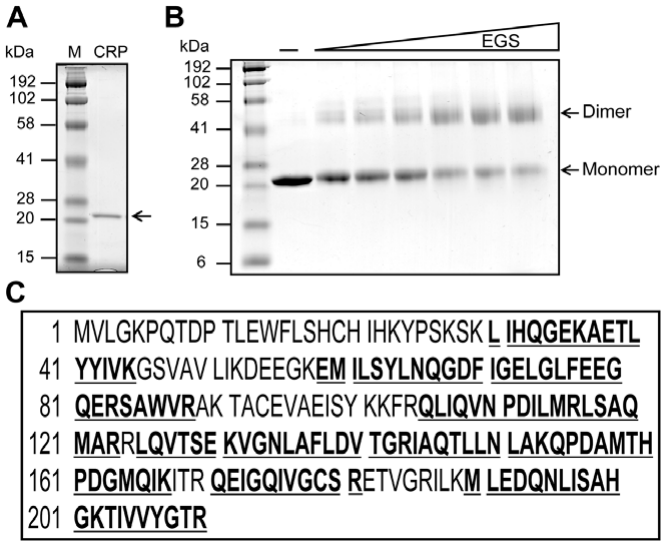
Expression and characterization of *E. coli* CRP. ***A***. SDS-PAGE profile of purified CRP. ***B***. Chemical cross-linking assays of purified *E. coli* CRP. The minus sign decodes no addition of the chemical cross-linker EGS, whereas the EGS concentrations were 1, 2, 5, 10, 15, and 20 µM. The cross-linking reaction mixtures were loaded on 12% SDS-PAGE. M denotes the Pre-stained broad range protein standards (BioRad). ***C***. MS identification of *E. coli* CRP protein. The tryptic peptides matching the CRP sequence are given in bold underlined type.

The global regulator cAMP-CRP complex [28] can act as either an activator [29] or a repressor [30] in expression of genes involved in many *E. coli* metabolic pathways. Although putative class I cAMP-CRP binding sites had been proposed upstream of some *fad* regulon genes and activation of *fadH* transcription was observed *in vivo* [17], direct physical evidence for DNA binding of these promoters by the cAMP-CRP complex was lacking. We therefore used EMSAs with purified CRP in the presence or absence of cAMP to test the proposed sites. SDS-PAGE analyses indicated that the apo-form of CRP of molecular weight ∼23 kDa was homogenous (Fig. 5A). Chemical cross-linking showed the protein was dimeric as previously reported [28] (Fig. 5B). Liquid chromatography mass spectrometry analysis of tryptic peptides demonstrated that our recombinant protein matched *E. coli* CRP with 66% coverage (Fig. 5C).

**Figure 6:**
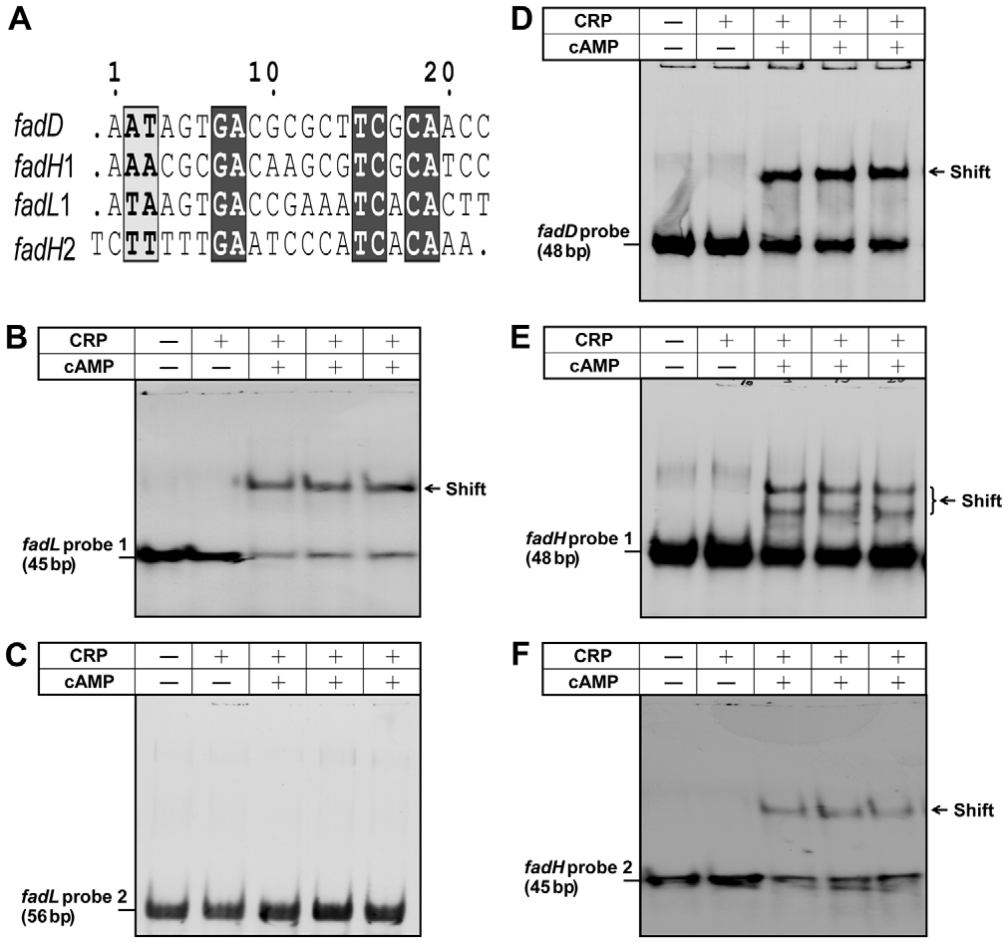
The cAMP-CRP complex binds the *fadL*, *fadD* and *fadH* promoters. ***A***. Sequence analyses of the CRP binding sites of *fadL*, *fadD* and *fadH*. White letters shaded in black denote strictly conserved bases, bold letters in grey represent highly similar residues, and dots mean gaps. The newly deduced CRP-binding site (*fadL*1 of *A*) binds the cAMP-CRP complex (panel ***B***) whereas the CRP-binding site predicted earlier [17] (abbreviated *fadL*2) does not (panel ***C***). *D* Binding of the *fadD* promoter region by the cAMP-CRP complex. ***E*** and ***F***. The *fadH* promoter region contains two functional CRP-binding sites, *fadH*1 and *fadH*2, respectively. The DIG-labeled probe shifted by the cAMP-CRP complex is indicated by an arrow. All EMSA experiments were carried out using 6.5% native PAGE and representative results are shown. The protein samples were incubated with 0.6 pmol of DIG-labeled probe in a total volume of 20 µl that contained 200 pmol cAMP (when added). The right hand four lanes of each contained (left to right) 10, 5, 10 and 20 pmol of CRP, respectively.

Higashitani and coworkers [4] predicted a *fadL* CRP binding site (Fig. 2B) centered at 57 bp downstream of the *fadL* transcription start site (termed *fadL*2) that is covered by our *fadL* probe 2, Fig. 6C). In contrast, we favored a site centered 157 bp upstream (Fig. 2C and 6A) covered by *fadL* probe 1 (Fig. 6B). In the *fadH* promoter, we previously proposed a CRP binding site (called *fadH* site 1 covered by *fadH* probe 1, Fig. 6A and E) and subsequently have predicted a second site (called *fadH*2 and covered by *fadH* probe 2, Fig. 6A and F). Given that sequence alignments of these sites showed only six conserved bp (Fig. 6A), the function of these sites required direct testing by EMSAs. As expected from numerous prior investigations (*e.g.*, Lawson *et al.* [31]), CRP lacked DNA binding activity in the absence of its cAMP ligand (Fig. 6). Gel shift assays confirmed that the CRP-cAMP complex efficiently bound *fadL* probe 1 (Fig. 6B), but failed to bind *fadL* probe 2 (Fig. 6C). Therefore, *fadL* site 1 is a functional CRP-cAMP complex binding site whereas the site predicted by Higashitani and coworkers [4] is nonfunctional. Unlike Class II sites which overlap the −35 hexamer and Class III sites which have tandem CRP binding sites, the position of *fadL* site 1 identifies it as an atypical site that is located too far upstream to be reached the C-terminal domain (CTD) of the α–subunit of RNA polymerase which seems to have maximum stretch of 90–120 bp upstream of the promoter. In contrast the demonstrated *fadD* CRP-cAMP complex binding site [27] can be clearly identified as a Class I site (Fig. 2C and 6D). Both proposed *fadH* promoter sites bound the CRP-cAMP complex and therefore comprise a bona fide Class III site of the type that is composed of two Class I sites (some Class III sites have a Class II site coupled to a Class I site) (Fig. 6E and F).

**Figure 7:**
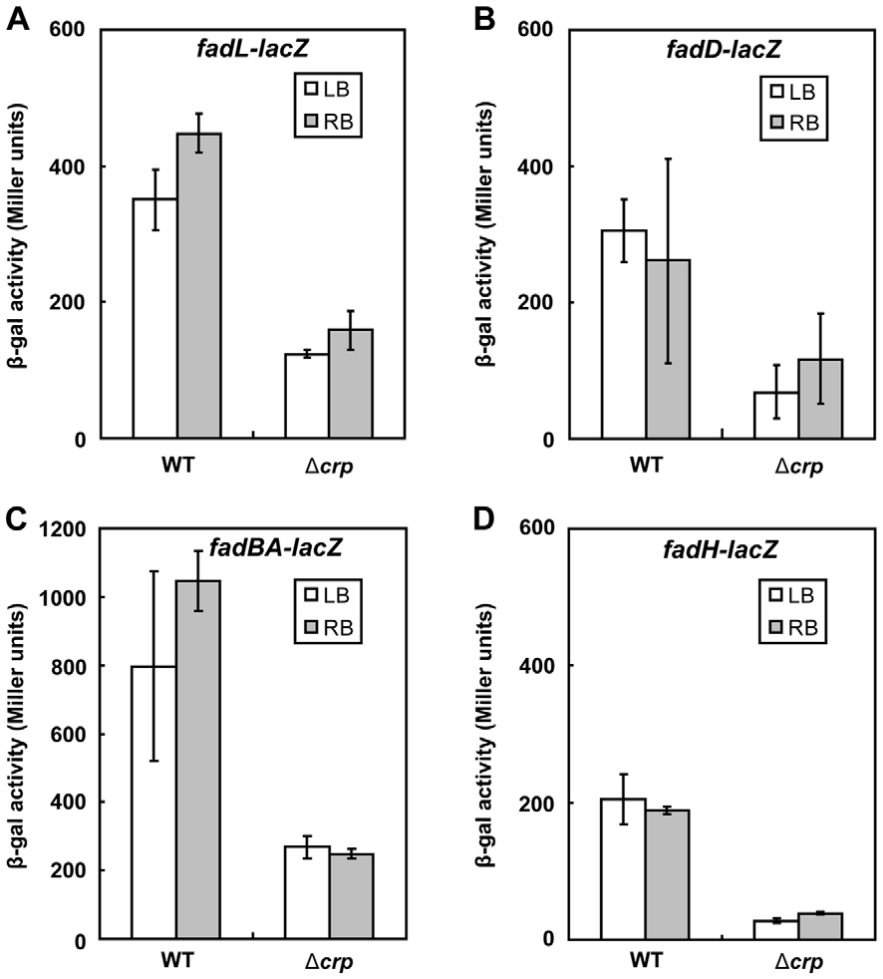
Activation of fatty acid transport system gene expression by the CRP-cAMP complex. ***A*** and ***B***. Decreased expression levels of *fadL* and *fadD*, respectively, were seen in the absence of CRP-cAMP. Strains FYJ104 (wild type) and FYJ134 (Δ*crp*) carry the *fadL-lacZ* transcriptional fusion whereas FYJ159 (wild type) and FYJ65 (Δ*crp*) contain the *fadD-lacZ* transcriptional fusion. ***C*** and ***D***. Expression of *fadBA* and *fadH* are positively regulated by the functional CRP-cAMP complex. Strains SI203 (wild type) and FYJ83 (Δ*crp*) carry the *fadBA-lacZ* transcriptional fusion whereas FYJ55 (wild type) and FYJ65 (Δ*crp*) contain the *fadH-lacZ* transcriptional fusion. The cultures were grown in either LB or RB media. beta-Galactosidase activities were from at least three independent experiments, and the error bars indicate standard deviations.

To evaluate the effects of CRP on expression of the fatty acid transport genes, *in vivo* assays of β-galactosidase activities of the *fadD* and *fadL lacZ* fusions in Δ*crp* mutant strains were compared to strains expressing wild type CRP (Fig. 7). As a control we also assayed *fadH* expression and found that expression decreased in the absence of CRP (Fig. 6E and F) (Fig. 7D). As expected, the activities of both transport promoters were modestly decreased upon the loss of *crp* (Fig. 7A and B) verifying that CRP activates *fadL* and *fadD* transcription. Although in agreement with others (http://www.ecocyc.org), we failed to identify any CRP-binding site candidates in the *fadBA* promoter, expression of *fadBA* in the *crp* deletion strain is decreased relative to the wild type strain (Fig. 7C). This may indicate an indirect activation of the *fadBA* promoter by the CRP-cAMP complex. It seems possible that the indirect activation is due to action of the FIS protein, expression of which is activated by CRP binding to three discontinuous sites [27]. Indeed, a putative FIS binding site (TTGCATATTTTTAACACAA, −36 to 18) lies within the *fadBA* promoter and FIS is a known activator of *fadBA* transcription [32].

### Expression of *fadL* and *fadD* are additively repressed by FadR and ArcA under anaerobic conditions

**Figure 8:**
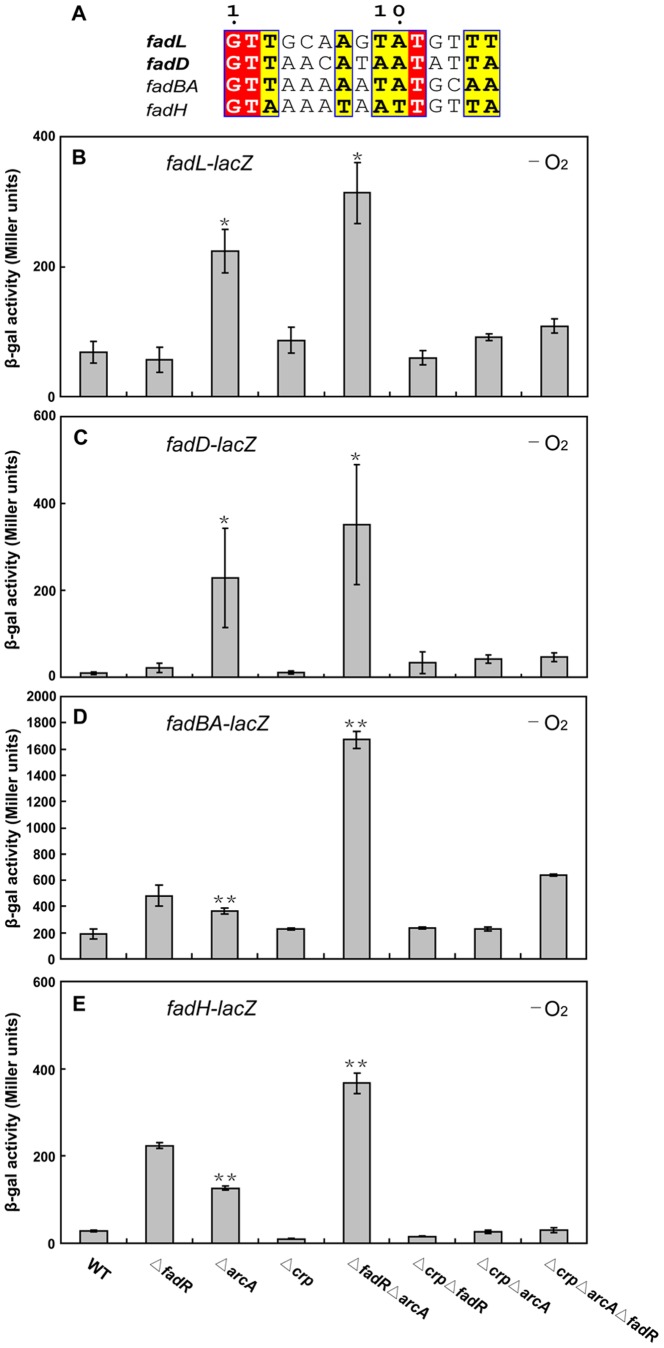
Negative regulation of *fadL* and *fadD* by ArcA-P under anaerobic conditions. ***A.*** Alignments of the ArcA-P binding sites of with those of other *fad* regulon genes. Red background indicates strictly conserved bases whereas yellow background denotes less conserved bases. ***B.*** Modulation of LacZ activity of the *fadBA-lacZ* transcriptional fusion by different regulatory proteins under anaerobic conditions. The *fadBA-lacZ* strains were SI203 (WT), SI207 (Δ*fadR*), FYJ82 (Δ*arcA*), FYJ83 (Δ*crp*), FYJ84 (Δ*arcA* Δ*fadR*), FYJ85 (Δ*crp* Δ*fadR*), FYJ86 (Δ*crp* Δ*arcA*) and FYJ87 (Δ*crp* Δ*arcA* Δ*fadR*), respectively. ***C.*** Transcriptional control of the *fadH-lacZ* fusion by different regulatory proteins under anaerobic conditions. The *fadH-lacZ* fusion strains were FYJ55 (WT), FYJ56 (Δ*fadR*), FYJ64 (Δ*arcA*), FYJ65 (Δ*crp*), FYJ68 (Δ*arcA* Δ*fadR*), FYJ81 (Δ*crp* Δ*fadR*), FYJ79 (Δ*crp* Δ*arcA*) and FYJ80 (Δ*crp* Δ*arcA* Δ*fadR*), respectively. ***D.*** Effect on *fadL* transcriptional levels by different regulatory proteins under anaerobic conditions. The *fadL-lacZ* were FYJ104 (WT), FYJ105 (Δ*fadR*), FYJ133 (Δ*arcA*), FYJ134 (Δ*crp*), FYJ141 (Δ*arcA* Δ*fadR*), FYJ142 (Δ*crp* Δ*fadR*), FYJ169 (Δ*crp* Δ*arcA*), and FYJ170 (Δ*crp* Δ*arcA* Δ*fadR*), respectively. ***E.*** Regulation of *fadD* transcription by different regulatory proteins under anaerobic conditions. The *fadD-lacZ* fusion strains were FYJ159 (WT), FYJ161 (Δ*fadR*), FYJ162 (Δ*arcA*), FYJ163 (Δ*crp*), FYJ164 (Δ*arcA* Δ*fadR*), FYJ165 (Δ*crp* Δ*fadR*), FYJ166 (Δ*crp* Δ*arcA*), and FYJ167 (Δ*crp* Δ*arcA* Δ*fadR*), respectively. The strains were grown on LB agar plates supplemented with 5 mM nitrate (KNO_3_) as the electron receptor. Anaerobic environments were generated using Bio-Bag environmental chamber type A as described [17]. beta-Galactosidase activities were recorded from at least six independent assays and are expressed as means ± standard deviations. *, P<0.005; **, P<0.001.

The weak effects of FadR inactivation on expression of *fadL* and *fadD* suggested that other regulatory proteins may be involved and thus we tested the effects of deletion of the genes encoding the other proteins proposed to bind these promoters. The oxygen-sensitive two-component system ArcA-ArcB was reported to negatively regulate transcription of several *fad* regulon genes, including *fadL*, *fadD*, *fadBA* and *fadH* [12]. That report [12] also contained *in vitro* and *in vivo* evidence that the phosphorylated ArcA regulator (ArcA-P) bound the promoters of the target genes (Fig. 8A). We recently reported cross-talk between FadR and ArcA in expression of other *fad* regulon genes and found that the interaction could be either additive (*fadH*) (Fig. 8E) or synergistic (*fadBA*) (Fig. 8D) [17]. The *fadD* promoter FadR site 2 and the ArcA site overlap by a single bp (Fig. 2C) as also seen in *fadH* [17] whereas in the *fadL* promoter the ArcA and FadR site 1 sequences are separated by 5 bp (Fig. 2B). Thus, cross-talk between FadR and ArcA in *fadL* and/or *fadD* expression seemed likely.

To test for cross-talk strains carrying *arcA* and/or *fadR* null mutations plus the chromosomal *fadL-lacZ* (or *fadD-lacZ*) transcriptional fusions were constructed. In general agreement with the report of Cho *et al.* [12], we found that the absence of *arcA* under anaerobic conditions resulted in increased transcription of *fadL* and *fadD* by 2 to 3-fold and >20-fold, respectively, (Fig. 8B and C). Our levels of derepression are somewhat less than those reported by Cho and coworkers [12] which we attribute to the gene used by these workers as their internal reference [17], [33]. However, our data differed from those of Cho and coworkers in the effects reported on FadR regulation of *fadL* transcription under anaerobic conditions when ArcA is functional (we see no effect versus their reported 4-fold increase). Although our statistical analysis argued that these two regulatory proteins control transcription of *fadL* and *fadD* in an additive manner, the wide variations in the data and the overlapping error bars provide caveats to this interpretation.

In the absence of the cAMP receptor protein (CRP) complex (cAMP-CRP), FadR and ArcA-ArcB repression of *fadL* and *fadD* expression was relatively weak (Fig. 8) and similar to that seen with *fadBA* and *fadH* [17]. Thus it seems that regulation by both FadR and ArcA-ArcB rely on the cAMP-CRP complex to activate transcription such that it can be further modulated.

### Physiological relevance of ArcA and the CRP-cAMP complex to fatty acid transport

**Figure 9:**
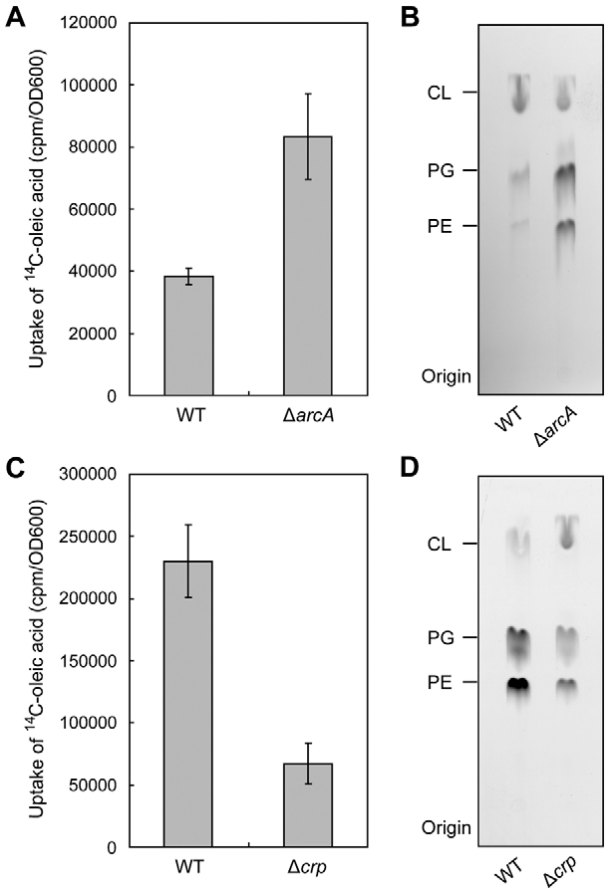
Effects of ArcA and CRP on incorporation of [1-^14^C]oleic acid into membrane phospholipid. ***A.*** Incorporation in the Δ*arcA* strain, FYJ82, and the wild type strain, SI203. At least three independent experiments were carried out and the data are expressed as mean ± standard deviation. ***B.*** A representative autoradiogram of a TLC separation of the ^14^C-labeled phospholipids of a panel ***A*** experiment. The phospholipid species are phosphatidylethanolamine (PE), phosphatidylglycerol (PG) and cardiolipin (CL). A representative autoradiogram is given. ***C.*** Incorporation in the Δ*crp* strain, FYJ83 and the wild type strain, SI203. At least five independent experiments were performed and the data are expressed in means ± SD. ***D.*** A representative autoradiogram of a TLC separation of the ^14^C-labeled phospholipids of a panel ***C*** experiment. Bacterial strains used in panel ***A*** and ***B*** were kept under anaerobic condition (details seen in Materials and Methods), whereas experiments in panel ***C*** and ***D*** were routinely conducted under aerobic condition.

Uptake of [1-^14^C]oleic acid was assayed to evaluate the physiological consequences of ArcA-ArcB and the CRP-cAMP complex on fatty acid transport (Fig. 9). To rule out potential interference by β-oxidation, the strains studied lacked *fadBA* (Table 1). Incorporation of [1-^14^C]oleic acid into the membrane phospholipids of the Δ*arcA* strain (FYJ82) was consistently >2-fold greater that that of its parental strain under anaerobic conditions (Fig. 9A). Further analyses by thin layer chromatography showed that incorporation into the three major membrane phospholipids phosphatidylethanolamine (PE), phosphatidylglycerol (PG) and cardiolipin (CL) (Fig. 9B), consistent with anaerobic repression of *fadL* (and/or *fadD*) by ArcA-P (Fig. 8B and C).

In agreement with the observed CRP-cAMP activation of *fadL* and *fadD* transcription (Fig. 7A and B), quantitative determination of total ^14^C-labeled bacterial membrane phospholipids showed that the level of oleic acid incorporation in the strain lacking *crp* is only about one third that of the parental strain (Fig. 9C and D). To our knowledge these are the first direct physiological data that directly show that these two global regulators modulate LCFA transport pathway in *E. coli*.

## Conclusions

Transcriptional regulation of fatty acid transport in *E. coli* involves three distinct regulatory systems, the specialized FadR system and the two global systems, ArcA and CRP-cAMP. FadR action is straightforward, it acts as a classical LacI-type repressor and only weakly represses *fadD* and *fadL* expression. CRP-cAMP regulation is also straightforward, fatty acids are a low status carbon source and *E. coli* prefers to use the highest status carbon source, glucose. In the presence of glucose (or in the absence of CRP), the other two regulators have little transcription to regulate. The apparent surprise is the stringent repression by ArcA seen under anaerobic conditions even in the absence of FadR because *E. coli* has a pathway to degrade fatty acids under anaerobic conditions [34] and low expression of FadD and FadL would seem likely to compromise function of the anaerobic pathway. However, under anaerobic conditions a new acyl-CoA synthetase, FadK, is induced that replaces FadD [35]. Unlike FadD which is inactive with short chain fatty acids [24], [35], FadK strongly prefers short chain length acids and such acids are the preferred growth substrates of the anaerobic β-oxidation pathway [34] although LCFA can also be utilized. Short chain acids readily enter *fadL* strains [36] and thus decreased expression of FadL is irrelevant for these growth substrates. However, degradation of LCFA such as oleate via the anaerobic pathway should require FadL. These apparent contradictions can be reconciled by our finding that ArcA represses *fadL* expression less than *fadD* expression (only about 4-fold) (Fig. 8B and C) and that FadK activates oleate poorly [35]. Hence, the significant level of FadL expressed under ArcA repression probably provides sufficient oleate transport to satisfy the poor catalytic activity of FadK with this substrate and allow the observed slow anaerobic growth on oleate [35].
